# Towards the Development of Novel Hybrid Composite Steel Pipes: Electrochemical Evaluation of Fiber-Reinforced Polymer Layered Steel against Corrosion

**DOI:** 10.3390/polym13213805

**Published:** 2021-11-03

**Authors:** Fatima Ghassan Alabtah, Elsadig Mahdi, Faysal Fayez Eliyan, Elsadig Eltai, Marwan Khraisheh

**Affiliations:** 1 Mechanical Engineering Program, Texas A&M University at Qatar, Doha 23874, Qatar; marwan.khraisheh@qatar.tamu.edu; 2 Department of Mechanical and Industrial Engineering, Qatar University, Doha 23874, Qatar; elsadigms@qu.edu.qa (E.M.); elsadig.eltai@qu.edu.qa (E.E.); 3 Engineering Technology Department, Community College of Qatar, Doha 23874, Qatar; faysal.eliyan@ccq.edu.qa

**Keywords:** fiber-reinforced polymer, pipeline, corrosion, steel, composite

## Abstract

Corrosion remains one of the major and most costly challenges faced by the steel industry. Various fiber-reinforced polymer coating systems have been proposed to protect metallic piping distribution networks against corrosion. Despite increasing interest among scientific and industrial communities, there is only limited predictive capability for selecting the optimum composite system for a given corrosive condition. In this study, we present a comprehensive evaluation of the electrochemical behavior of two different fiber-reinforced polymer composite systems against the corrosion of carbon steel pipes under a wide range of acidic and corrosive solutions. The composites were made of glass and Kevlar fibers with an epoxy resin matrix and were subjected to corrosive solutions of 0.5 M NaCl, 0.5 M HCl, and 0.5 M H_2_SO_4_. The kinetics of the corrosion reactions were evaluated using potentiodynamic polarization (PDP) tests. In addition, electrochemical impedance spectroscopy (EIS) tests were carried out at open circuit potentials (OCPs). It was demonstrated that the glass fiber-reinforced polymer coating system offered the best protection against corrosion, with a high stability against deterioration when compared with epoxy and Kevlar fiber-reinforced polymer coating systems. Scanning electron microscopy images revealed cracks and deteriorated embedded fibers due to acid attack, sustained/assisted by the diffusion of the corrosion species.

## 1. Introduction

Corrosion is considered to be the leading cause of failure in pipelines transporting gases and liquids [1]. The primary mandate of pipeline designers is to cope with the rapid increase in requests for energy while reducing the cost and environmental impacts [2,3,4]. Nowadays, metallic and composite pipelines are the most cost-effective way of transporting water, oil, and gas [5]. Low-to-moderate pressure composites and plastic flow line and gathering line systems for oil and natural gas have been in service for many years. However, the existing oil and gas pipeline technology cannot be extrapolated to successfully achieve the required cost and performance goals needed in order to implement an extensive distribution network. The conventional metallic and composite material-based pipelines widely used in the steel industry are likely to experience catastrophic failures due to corrosion and abrasion [6]. Corrosion degrades the strength capacity of metallic pipelines, while matrix cracking/abrasion causes the leakage of composite pipelines [7]. Both corrosion and abrasion cause significant losses and decrease the structural integrity of pipelines [8]. Corrosion in metallic pipelines can be classified into two categories: external and internal. External corrosion is generally controlled using organic coatings and cathodic protection, while corrosion inhibitors are widely used to fight internal corrosion [9,10]. Various coating types have been used to protect pipeline surfaces against corrosion [11]. However, some problems such as pores, cracks, scratches, and other types of damage limit the effectiveness of coatings as protective barriers, exposing the surface to corrosive environments [12]. The polymeric coating degradation process could involve a micro-separation process with the enlargement of sub-molecular structures, leading to deficient coating systems [13]. The utilization of epoxy coatings, especially in high-performance applications, is limited to high rigidity and low-impact strength despite efforts to achieve enhancements in toughness with a minimal loss of mechanical properties [14]. Experts in the oil and gas industries agree that future transmission pipelines will operate at higher pressures and will be more resistant to corrosion [15]. To meet the increased demands, maintain safety and reliability, and be competitive, pipeline designers and operators are looking for alternative materials and composite systems to conventional metallic pipes and pure composite pipelines [16]. One obvious alternative is to use fiber-reinforced polymers (FRPs) as a coating for metallic pipes. Previous investigations have indicated that the use of FRP composite materials to protect steel pipes is efficient against corrosion, cost effective, lightweight [17,18], and imparts extra strength and internal pressure capacity to the pipe [19,20,21]. FRP composite materials are widely used in the petroleum and chemical industries [22]. Despite their effectiveness against corrosion, long-term exposure to harsh environmental conditions such as extreme temperatures and humidity, UV light, and different acidic environments negatively affects FRP composites’ performance and durability [23,24,25]. Recent investigations have reported considerable differences between the properties of initial and aged FRP specimens [26,27,28,29,30]. FRP material degradation is a significant issue with economic consequences [31,32].

Furthermore, the degradation influences the composite components—namely, the matrix, the fibers, and the interface [33,34]. For this reason, the search for the optimum composite coatings for a specific environment and operating conditions is ongoing, and several investigators are working on this topic. There is an urgent need to develop guidelines (or standards) accepted by both the scientific and industrial communities to select optimum composite coatings for metallic pipes that have been customized for the desired operating conditions. A critical step towards this goal is a comprehensive study of the corrosion and electrochemical behavior of the most effective FRPs under various corrosive solutions. In this study, we evaluate the electrochemical behavior of FRP systems against the corrosion of carbon steel pipes under various acidic and corrosive solutions. Two different fiber types were investigated: glass and Kevlar fibers with an epoxy resin matrix. Glass and Kevlar fibers are economical and commonly used as reinforcements because of their excellent mechanical and insulating properties and resistance to deterioration [35,36].

To highlight the importance of this study, the electrochemical behavior of different coatings and FRP systems as anti-corrosive coatings for carbon steel (CS) in different solutions and corrosive mediums reported in recent literature are summarized in Table 1. The existing studies that have used a carbon fiber-reinforced polymer (CFRP) have indicated that galvanic corrosion generally occurs when steel is mechanically joined to a CFRP material in aggressive environments. This is why the CFRP was not chosen as an FRP system in the current investigation, whereas GFRP and KFRP were chosen due to their superior properties. Few studies covering the electrochemical evaluation of FRP materials in oil and gas pipeline environments were found. Further studies on the electrochemical behavior of different FRP systems in different corrosive environments should be conducted to increase our confidence in expanding the market using this promising material system, which was the scope of this study.

## 2. Materials and Methods

### 2.1. Materials

Cleaned EN10130 carbon steel sheet specimens with a thickness of 1.5 mm, length of 8 cm, and width of 4 cm were used as the substrate for epoxy coatings and FRP layers. The chemical composition of the EN10130 carbon steel sheet is given in Table 2. First, the specimens were ground using different abrasive papers, up to 1200 grits, to improve the epoxy and FRP layer adhesion with the steel surface and prevent delamination. Next, the specimens were washed with water, degreased in acetone, washed again, and dried with air.

**Figure 1:**
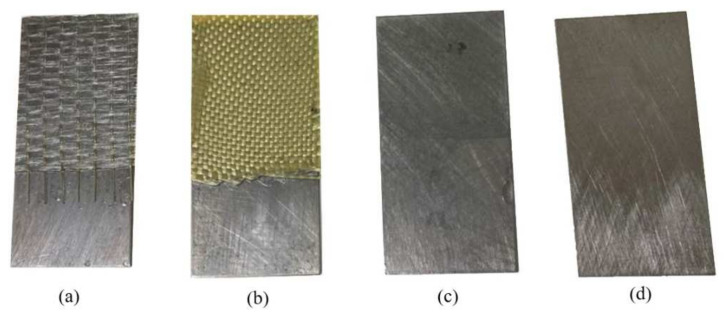
Prepared specimens: (**a**) GFRP/steel, (**b**) KFRP/steel, (**c**) epoxy/steel, (**d**) non-coated steel.

**Figure 2:**
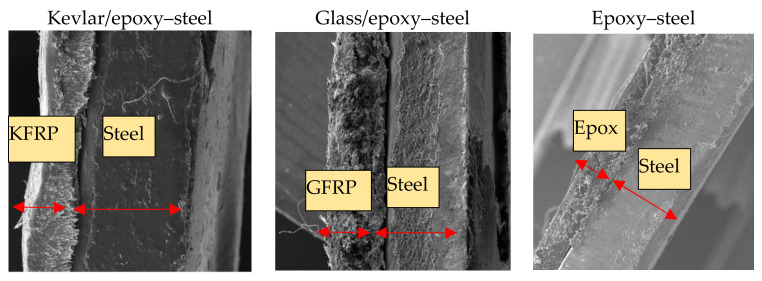
Cross-sectional SEM images of the prepared specimens.

Four types of specimens were prepared using the hand layup process, as shown in Figure 1. The first type is a control sample, a non-coated steel specimen referred to as the exposed substrate. The second type is the epoxy-coated steel specimen, referred to as epoxy/steel. The third and fourth types are the glass fiber-reinforced polymer and Kevlar fiber-reinforced polymer layers added to the steel specimens, referred to as GFRP/steel and KFRP/steel, respectively. The glass fabric (grade GF-22-280-100) and Kevlar fabric (grade K-22-300-100) are (0°/90°) woven fabrics manufactured by Easy Composites Ltd. The polymeric matrix comprises 100 parts epoxy resin (EL2) and 30 parts curing agent (AT30). The prepared specimens were left for 48 h at room temperature to cure. The average thickness of the epoxy coating and FRP composite layers in all the specimens was 0.5 mm for each layer. Figure 2 shows the SEM images of the cross-sections of the prepared specimens.

### 2.2. Methods

#### Potentiodynamic Polarization (PDP) and Electrochemical Impedance Spectroscopy (EIS)

**Figure 3:**
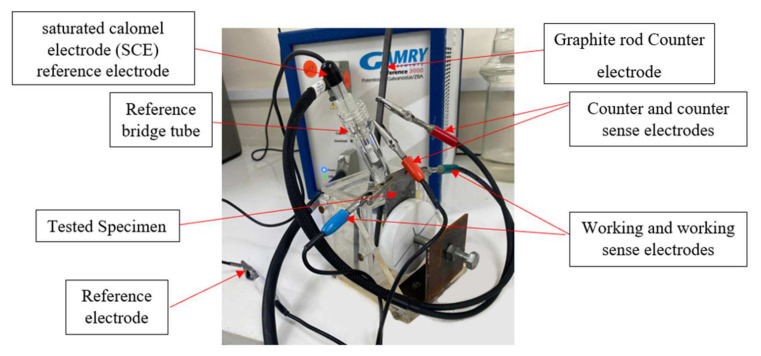
The electrochemical cell setup.

Potentiodynamic polarization testing was used to evaluate the kinetics of the corrosion reactions and the corrosion rates and potentials concerning the anodic reactions, the cathodic reactions, and passivity [43]. The potentiodynamic polarization scans were from −1.2 V to 0.5 V (SCE). In addition, EIS was used to quantify the impedance of coatings to corrosion and to evaluate the interfacial interactions of the coated systems from the solution interface, across the coating, and near the steel substrate, according to the ASTM-G106 standard [44]. The four specimens were immersed in 0.5 M NaCl, 0.5 M HCl, and 0.5 M H2SO4 solutions to study their behavior. An EIS study was carried out at open circuit potentials (OCPs) within the frequency range of 0.2 Hz to 100,000 Hz. OCP tests were carried out to evaluate the dominance of the anodic reactions versus the cathodic reactions and their variations with time concerning the coating type system and the environmental conditions. The electrochemical tests were carried out with the GAMRY 3000 potentiostat, as shown in Figure 3.

## 3. Results and Discussion

### 3.1. OCP/Potentiodynamic Polarization

**Figure 4:**
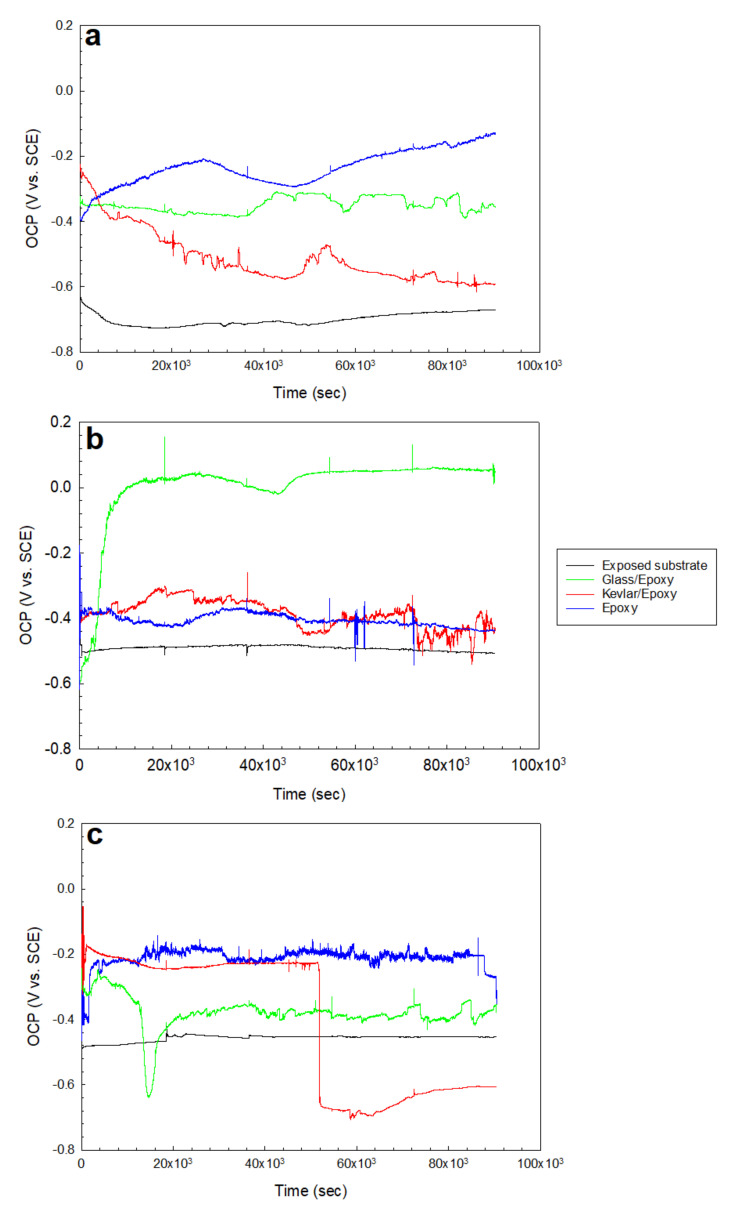
Open-circuit potentials in (**a**) 0.5 M NaCl, (**b**) 0.5 M HCl, and (**c**) 0.5 M H_2_SO_4_ solutions for the exposed substrate and glass/epoxy, Kevlar/epoxy, and epoxy systems.

**Figure 5:**
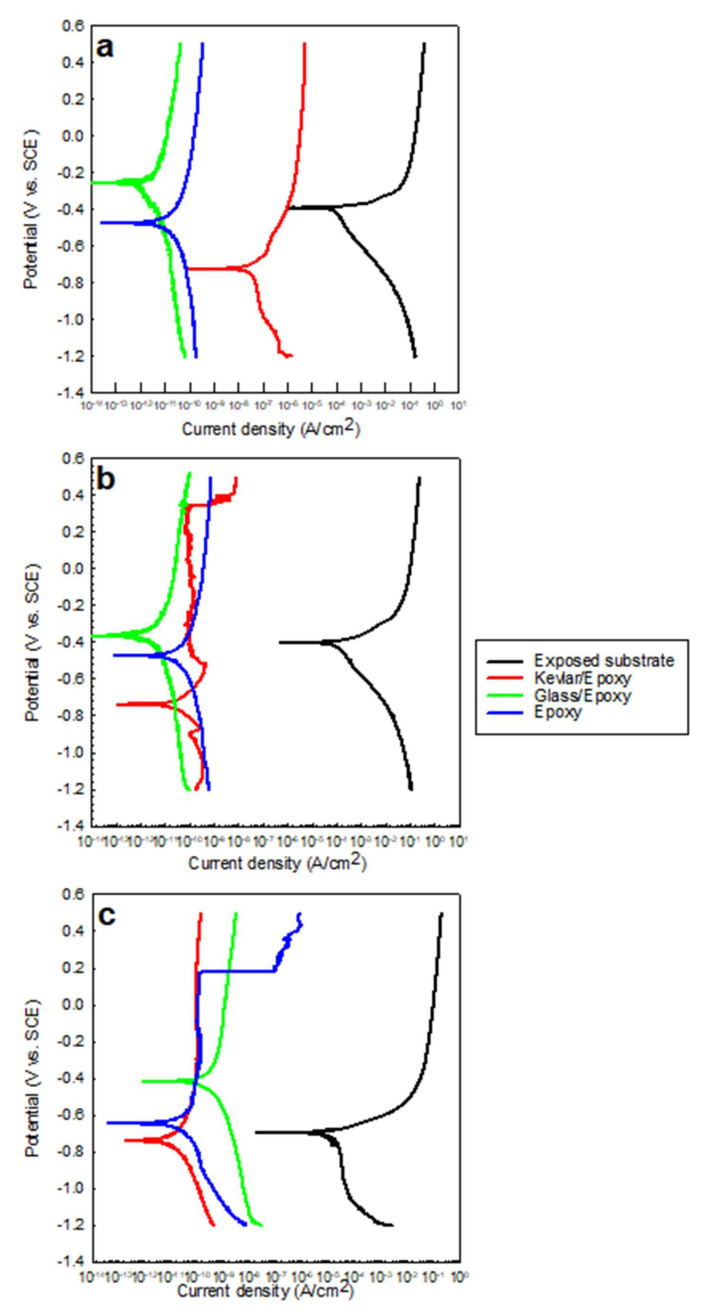
Potentiodynamic polarization in (**a**) 0.5 M NaCl, (**b**) 0.5 M HCl, and (**c**) 0.5 M H_2_SO_4_ solutions for the exposed substrate and glass/epoxy, Kevlar/epoxy, and epoxy systems.

**Figure 6:**
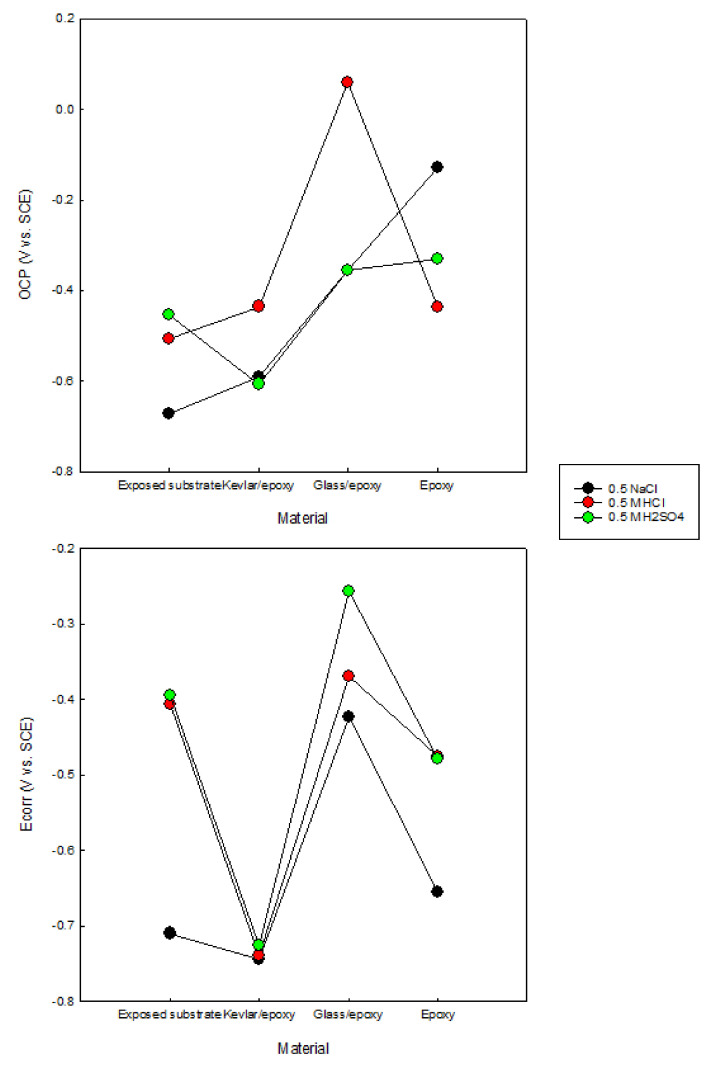
Open-circuit and corrosion potentials in 0.5 M NaCl, 0.5 M HCl, and 0.5 M H_2_SO_4_ solutions for the exposed substrate and glass/epoxy, Kevlar/epoxy, and epoxy systems.

The samples; the exposed substrate; and the glass/epoxy, Kevlar/epoxy, and epoxy systems were immersed for 25 h in the testing solutions of 0.5 M NaCl, 0.5 M HCl, and 0.5 M H_2_SO_4_. The open-circuit potentials were monitored (including the electrochemical impedance spectroscopy, with the timings outlined in Section 3.2). In addition, potentiodynamic polarization scans were carried out at the end of immersion. This test evaluates the corrosion resistance, corrosion kinetics, passivation, and cathodic reactions concerning a coating system and testing solution. The tests offered findings on the feasibility of a coating type in mild and severe environmental conditions. The OCPs, as shown in Figure 4, indicate the noticeably decelerated anodic activity of the substrate when coated, for higher OCPs than the exposed substrate in all states. The glass/epoxy system exhibited better corrosion/deterioration resistance in the acidic solutions than the Kevlar/epoxy system. In Figure 4c, the Kevlar/epoxy system interestingly shows deterioration in the H_2_SO_4_ solution to exhibit OCPs comparable to an exposed substrate after nearly 50 ks. The potentials dropped to nearly −0.6 V vs. SCE, below the steady potential of nearly −0.48 V vs. SCE, indicating the vigorous anodic activity sustained by H_2_SO_4_. The epoxy alone seemed to demonstrate corrosion resistance intermediate between the Kevlar/epoxy and glass/epoxy systems. The potentiodynamic scans of the coated systems in Figure 5 indicate a comparable polarization response in the anodic reactions, cathodic reactions, and passivation. No one solution showed a more significant influence than the others, and no coating system showed a more significant corrosion resistance. The corrosion rates were 1 million to 10 million times less than those of the exposed substrates. In agreement with the OCPs, the glass/epoxy system had high corrosion potentials. The Kevlar/epoxy system had a low corrosion potentials, indicating the decelerated anodic activity and the effectiveness of the glass/epoxy system. In the long run, in acidic conditions glass promotes and Kevlar lessens corrosion resistance. At the end of the experiments in acidic conditions, the Kevlar/epoxy layer showed a mild dissociation. The relative agreement between the OCP and Ecorr values is shown in Figure 6. The high Ecorr values of the exposed substrate in acidic conditions result from the high rate of cathodic reactions. It should be noted from Figure 5 that, during passivation, unlike the glass/epoxy system the epoxy alone and epoxy/Kevlar systems showed instability manifested as the abrupt increase in passive currents between 0.2 and 0.4 V vs. SCE in the acidic solutions.

### 3.2. Electrochemical Impedance Spectroscopy (EIS)

The electrochemical impedance spectroscopy response was monitored across periods of 5, 10, 15, 20, and 25 h of free immersion in 0.5 M NaCl, 0.5 M HCl, and 0.5 M H_2_SO_4_ solutions for the steel surface and surfaces coated with glass/epoxy, Kevlar/epoxy, and epoxy systems. The purpose of this was to evaluate (i) the nature of the interfacial interactions on the protective epoxy-based systems, across them, and beneath them at the steel/layer interface; (ii) the mechanisms of corrosion and mass transport; (iii) the deterioration of the epoxy or FRP; (iv) the distribution of the anodic and cathodic reactions with time, and concerning the environmental conditions and physical properties of the layer system, and in comparison to the response of a steel substrate without protection. In addition, the results were used to corroborate the kinetic results of the OCP and potentiodynamic polarization. Regardless of the solution (medium-pH (0.5 M NaCl) or low-pH (0.5 M HCl and 0.5 M H_2_SO_4_)), the anodic reaction involves the dissolution of the steel substrate, forming ferrous ions. Those ions migrate and accumulate first within possible pores reacting with hydroxide and O2 to form iron hydroxide (Fe(OH)_2_), iron carbonate (FeCO_3_), and iron oxides (Fe_2_O_3_ and Fe_3_O_4_) [45]. However, the anodic reaction proceeds at different rates depending on the pH and the nature of the interfacial protection used. The dissolution might remain underneath the protective layer because of the partial, with-time pore-like deterioration of the epoxy, causing the immersion solution to reach the substrate. Alternatively, it results in corrosion products that fill the pores, further weaken the epoxy (being weakened already from the solution), accumulate out of the epoxy layer, and partially cover it. These drawbacks affect the corrosion protection by suppressing both the anodic and cathodic reactions. They also affect the distribution of the anodic versus the cathodic reactions with time, changing the overall mechanism that controls the interfacial interactions. The significance of the cathodic reactions that involve hydrogen reduction is much higher in the HCl and H_2_SO_4_ solutions than in the NaCl solutions. The anodic dissolution with time becomes high as a result.

Nyquist and Bode plots were used to elucidate the interactions and their physical effects on the substrate and protective layer with time. It was determined that in all of the solutions, regardless of the physical conditions at the interface (bare surface or coated), the interactions proceeded with the exact mechanism and in a time-independent fashion. The equivalent circuit of the configuration, {R(Q(R(QR)))}, was correctly fitted to the experimental data across the entire frequency range, from the high frequency (charge transfer and surface interface) to the low frequency (epoxy layer and bulk solution). The suitability of the equivalent circuit to the bare steel surface related interestingly to the developing corrosion products, which with time achieved a significance similar to that of the original protective layer. To account for the heterogeneities, the capacitance of the double layer, corrosion products, and coating system was calculated as a constant phase element (CPE), with admittance expressed as [46].

(1) $${Y\;=\;Y}_{Q}{\;\omega }^{n}cos\left(\frac{n\pi }{2}\right){\;+\;jY}_{Q}\omega^{n}sin\left(\frac{n\pi }{2}\right)$$

where *ω* is the angular frequency and *n* is the CPE exponent.

**Figure 7:**
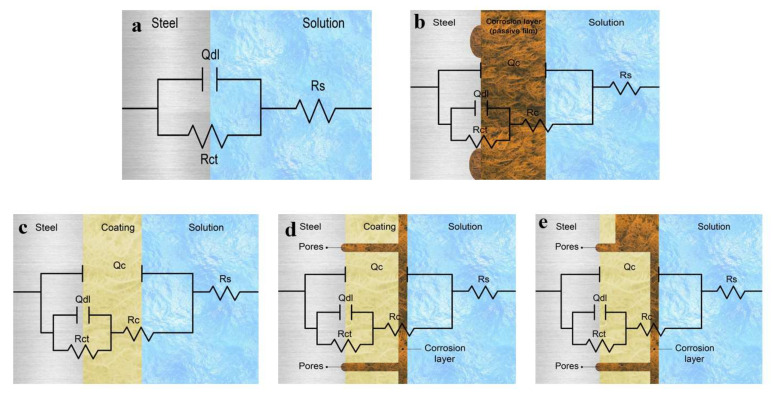
Electrochemical equivalent electric circuit models obtained from fitting the impedance data: (**a**) bare steel before corrosion, (**b**) corroded bare steel, (**c**) coated steel, (**d**) enhanced corrosion resistance, and (**e**) decreased corrosion resistance.

The equivalent electrical circuits were fitted using the Gamry Echem Analyst software. Figure 7 presents the electrochemical equivalent electric circuit models, fitting the impedance data of the bare and coated steel specimens. In this figure, Rs is the solution resistance, Rc is the coating resistance, Rct is the charge transfer resistance, Qc is the coating capacitance, and Qdl is the double-layer capacitance.

#### 3.2.1. EIS in 0.5 M NaCl Solutions

**Figure 8:**
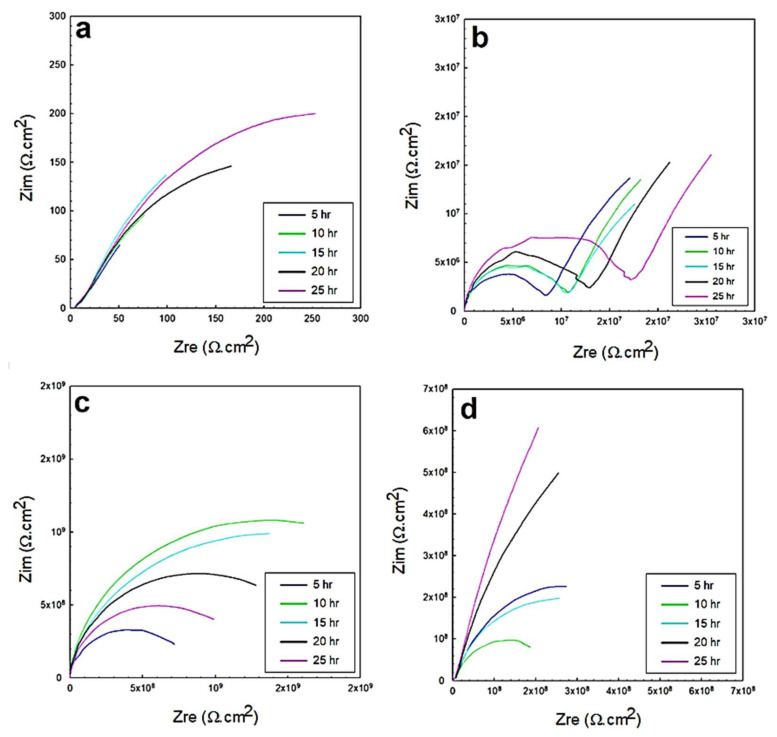
EIS Nyquist plots of the 0.5 M NaCl solution for: (**a**) exposed steel, (**b**) glass/epoxy, (**c**) Kevlar/epoxy, and (**d**) epoxy systems.

**Figure 9:**
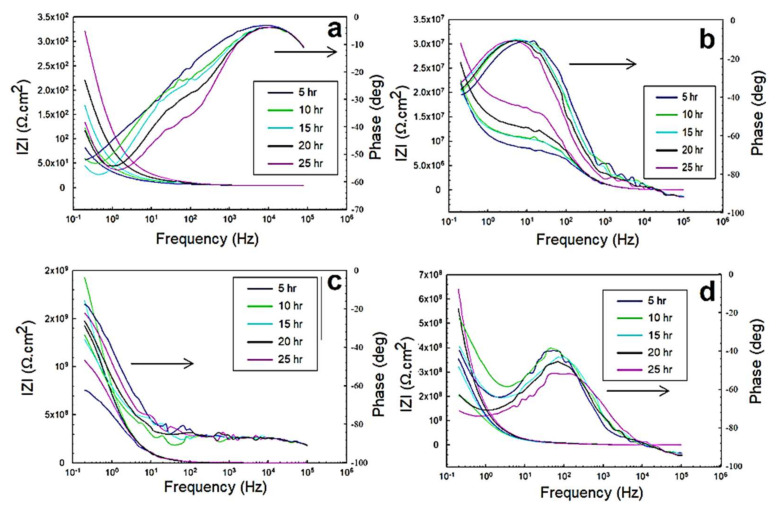
EIS Bode plots of the 0.5 M NaCl solution for: (**a**) exposed steel, (**b**) glass/epoxy, (**c**) Kevlar/epoxy, (**d**) epoxy systems.

The EIS response represented by the Nyquist and Bode profiles in the 0.5 M NaCl solution is shown in Figure 8 and Figure 9, corresponding to the exposed steel, glass/epoxy, Kevlar/epoxy, and epoxy systems. From the fitting of the data with the equivalent circuit, the interactions with time did not change in the governing mechanism. In addition, the interactions were independent of the nature of the coating system used. The behavior change was primarily governed by the changes the epoxy systems underwent and the evolution of the corrosion products in affecting the charge transfer resistance at the double layer and corrosion resistance of the protective layer. In the glass/epoxy system, the corrosion resistance increased from nearly 8 MΩ·cm^2^ to 16 MΩ·cm^2^, indefinitely with time. The admittance did not change, at nearly 190 × 10^−9^ S.sn/cm^2^, with exponent *n* values of nearly 0.7 at the double layer. This indicated the significance of the passive film in enhancing the protectiveness, in a pseudocapacitive manner, with pores continuously filled with protective film. The Kevlar/epoxy system, in agreement with the OCP and polarization data, showed the lowest corrosion resistance, which was decreasing with time, from nearly 6.5 MΩ·cm^2^ to 30 kΩ·cm^2^, with a higher admittance (that did not change with time) of nearly 150 × 10^−12^ S.sn/cm^2^. This underlines the deterioration of the Kevlar-based epoxy layer, thinning for greater permeation of the chloride and hydroxyl ions to accelerate the deterioration of the layer and dissolution of the substrate, leading to the formation of porous passive films. The epoxy-only layer exhibited a continuously decreasing corrosion resistance before it increased at the end of testing, indicating its reliability against corrosion in the long run without interference from glass or Kevlar. It had the lowest permeability value as it was across the double layer, and the epoxy layer was more capacitive than the glass/epoxy and Kevlar/epoxy systems. From the Bode diagrams, the phase peak values did not change, nor did they shift in frequency with time, indicating the single-phase constant response and similarity in the governing mechanisms of interactions, similar to the findings in [37,47]. The peaks of the glass/epoxy and epoxy systems indicated reliability against activity at both the double and coating layers, appearing at frequencies of two orders of magnitude higher than those of the Kevlar/epoxy system. In comparison, the EIS response of the steel substrate was more capacitive, two-time-constant-based, and of much lower resistance at the double and passive film layers (whose resistance increased with time). This result outlines the superior advantage of the coating systems utilized in the chloride solutions.

#### 3.2.2. EIS in 0.5 M HCl and 0.5 M H_2_SO_4_ Solutions

**Figure 10:**
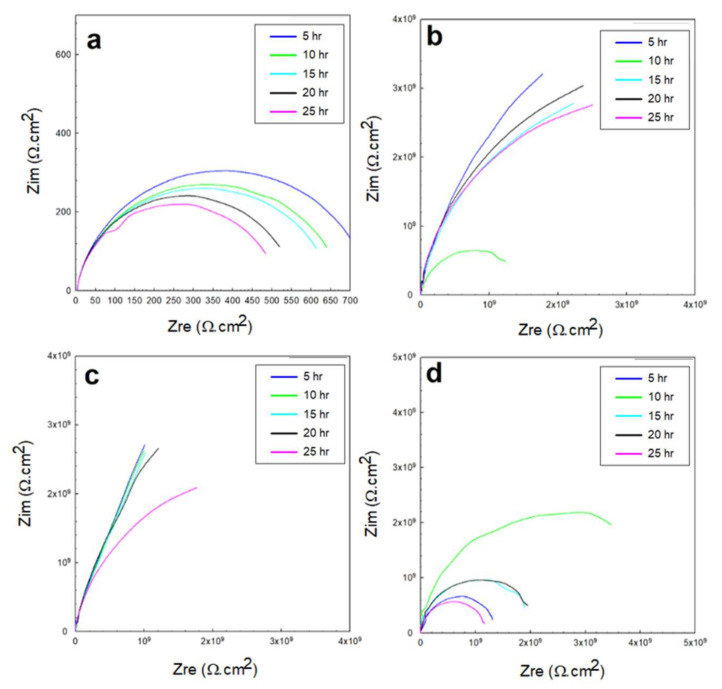
EIS Nyquist plots of the 0.5 M HCl solution for: (**a**) exposed steel, (**b**) glass/epoxy, (**c**) Kevlar/epoxy, and (**d**) epoxy systems.

**Figure 11:**
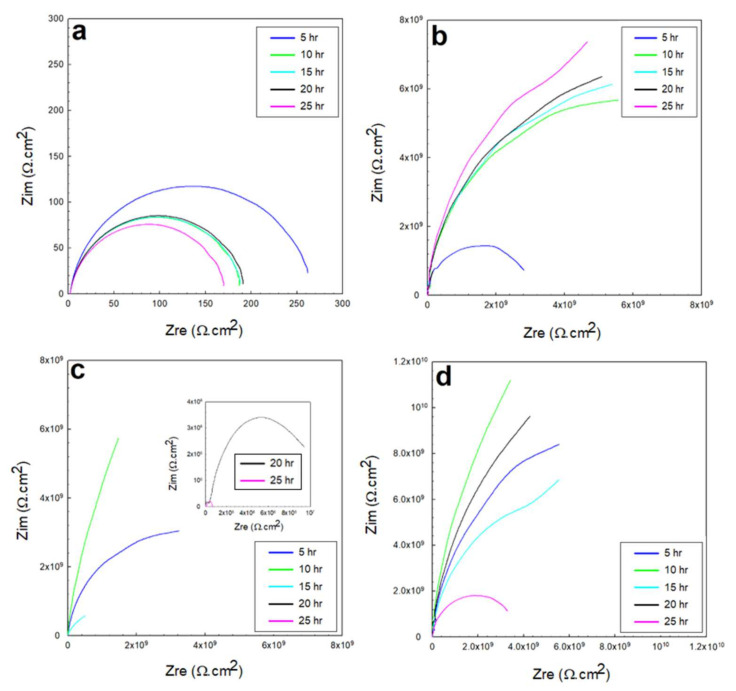
EIS Nyquist plots of the 0.5 M H_2_SO_4_ solution for: (**a**) exposed steel, (**b**) glass/epoxy, (**c**) Kevlar/epoxy, and (**d**) epoxy systems.

**Figure 12:**
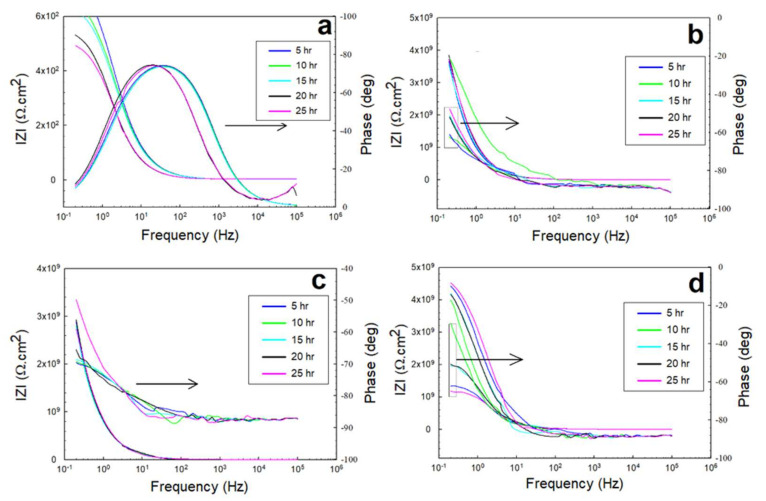
EIS Bode plots of the 0.5 M HCl solution for: (**a**) exposed steel, (**b**) glass/epoxy, (**c**) Kevlar/epoxy, and (**d**) epoxy systems.

The EIS response underwent the exact same governing mechanism of interfacial interactions in the acidic solutions as the one that governed the coated systems in the NaCl solutions (of mild pH). The dominance and rate of the cathodic reactions for the direct reduction of hydrogen protons onto and across the diffusion channels of the coated systems were higher at the beginning of the immersion. However, with time, this incentivized the anodic dissolution, passivation, and partial weakening or partial deterioration of the coating layer. The Nyquist profiles of the 0.5 M HCl and 0.5 M H_2_SO_4_ solutions shown in Figure 10 and Figure 11 indicate the relatively higher capacitance of the interactions across the low-frequency and high-frequency ranges. The variations in the overall capacitance in the two solutions with time reflected the competitive, opposing kinetics of the hydrogen evolution and the formation of passive films. In the HCl solutions, the resistance of the glass/epoxy system was the highest, nearly 7 GΩ·cm^2^, although showing a steady decrease with time to as low as 0.2 MΩ·cm^2^. The Kevlar/epoxy system, on the contrary, had less resistance. However, it increased with time from 45 kΩ·cm^2^ to 0.5 MΩ·cm^2^ and to 4 MΩ·cm^2^, underlining the significance of Kevlar in promoting the formation of passive films that, with time, decelerate dissolution and prove the significance of glass in stabilizing the epoxy system that initially protects the system. Interestingly, the epoxy-only system did not show a net change in resistance; it increased only after 10 h to decrease steadily back to nearly the initial resistance values of the charge transfer and protective layer at nearly 1.2 MΩ·cm^2^. Thus, the epoxy-only layer exhibited comparable reliability to that of the glass/epoxy layer against corrosion during the total time of the experiment. Furthermore, the phase peaks in Figure 12 indicate that the single-time-constant-based interactions were similar regardless of the coating systems, with peaks associated with interactions in the hydrodynamic layers away from the coating system at low frequencies, similar to the findings in [48].

**Figure 13:**
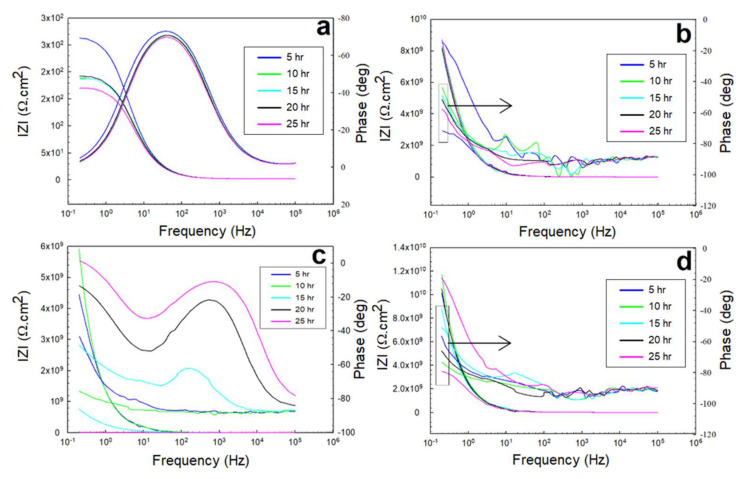
EIS Bode plots of the 0.5 M H_2_SO_4_ solution for: (**a**) exposed steel, (**b**) glass/epoxy, (**c**) Kevlar/epoxy, and (**d**) epoxy systems.

In H_2_SO_4_ solutions, the hydrogen generation was more vigorous. However, for a glass/epoxy system, it incentivized the anodic reactions to form protective films at rates high enough to surpass the disruption from the hydrogen generation, precipitate and decelerate dissolution, and promote the resistance of the layer. The resistance was higher than that of the HCl solutions, and it increased with time from nearly 3 MΩ·cm^2^ to 10 MΩ·cm^2^. In the Kevlar/epoxy system, however, the resistance was expectedly lower, and it decreased with time. The epoxy deteriorated with Kevlar fibers, not facilitating precipitation of the influential passive films on the heavily attacked surface with a system resistance of nearly 0.1 MΩ·cm^2^. Its Bode phase peaks, as shown in Figure 13, indicate the two-time-constant-based nature of the interactions, as reported in [49]. The epoxy-only system exhibited reliability better than the Kevlar/epoxy system, and the resistance decreased steadily with time to nearly 6 MΩ·cm^2^. The physical properties of the reinforcing elements of glass and Kevlar, if associated with the adherence of the passive films in acidic conditions in future studies, could be used to better predict the reliability of coating systems in the long run.

### 3.3. Failure Modes

#### 3.3.1. Optical Microscope Images

**Figure 14:**
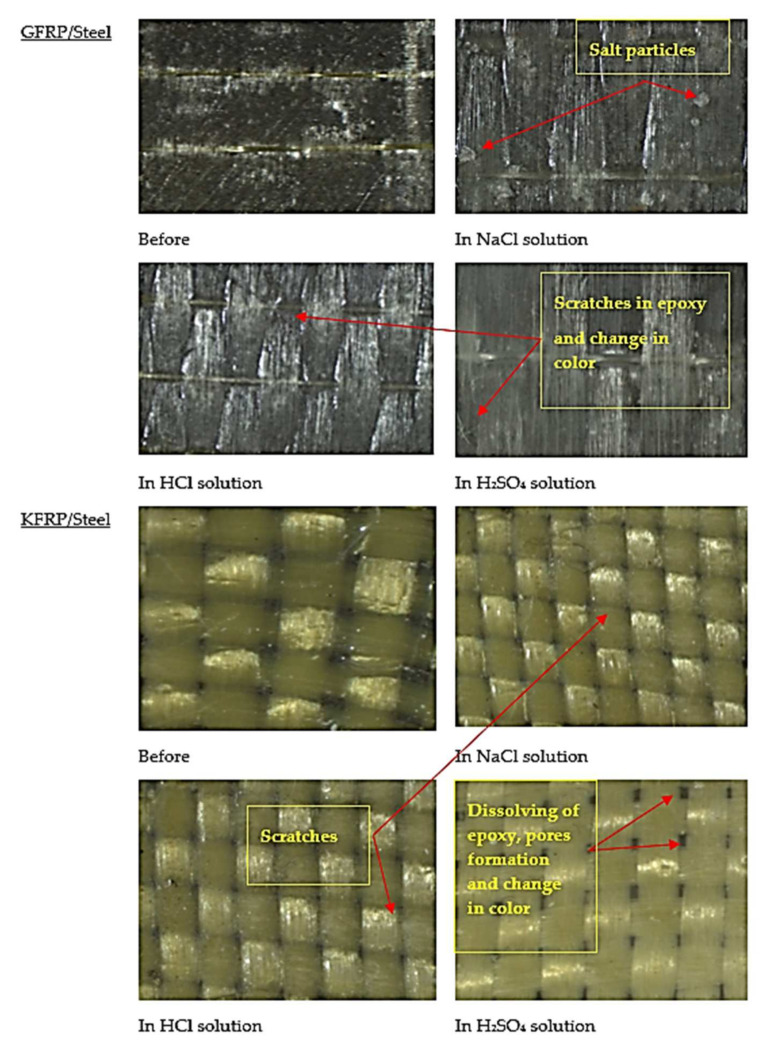

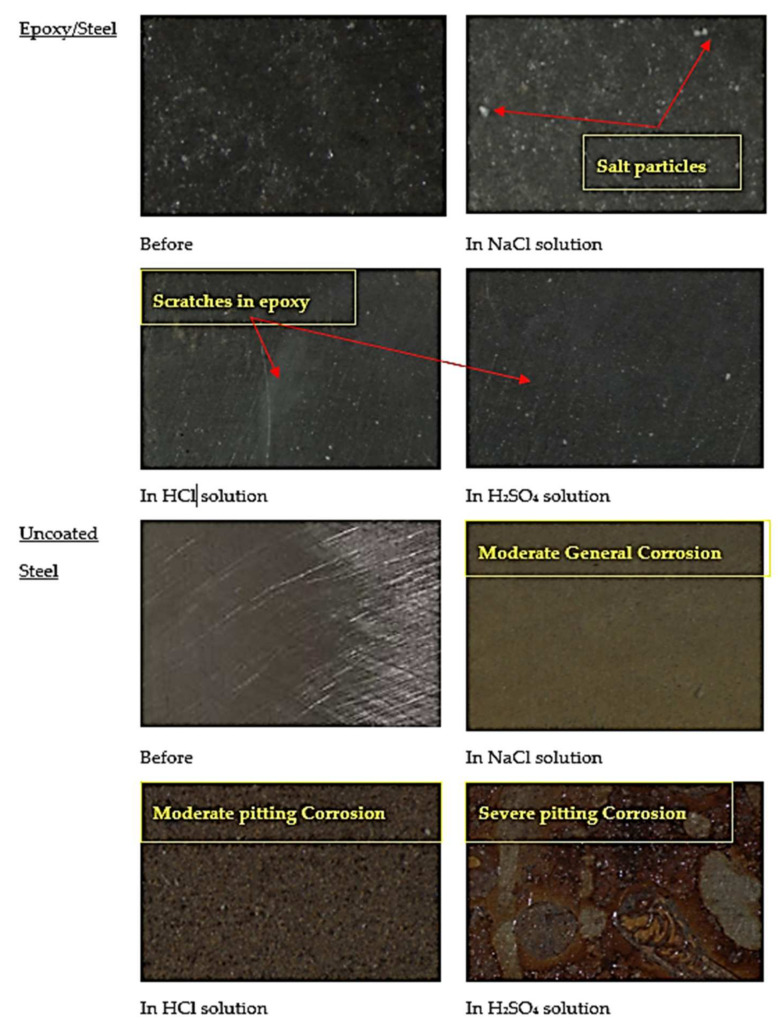
Optical microscope images for the tested specimens before and after the electrochemical testing.

Figure 14 presents optical microscope images of the tested samples before and after testing, and the failure mode of each specimen is described. The glass/epoxy system showed a high stability against corrosion in all solutions without signs of deterioration, pores, or significant localized disbandment or scratches through which sustained corrosion or mass transport could occur. Expectedly, the NaCl solution sample retained salt crystals. There was no evidence that chloride was involved in the localized corrosion—otherwise, iron chloride products would have intermixed with iron oxides. In the acidic HCl and H_2_SO_4_ solutions, slight localized changes in color were observed on the fibers. Kevlar samples showed deterioration in epoxy, and the appearance of pores and the color change was mostly in the H_2_SO_4_ solution. The epoxy-only system showed evidence of minor pores acting as diffusion channels, but it showed stability regardless of the environmental conditions. Comparably, the steel samples were heavily attacked, most severely in the acidic solutions.

#### 3.3.2. SEM Analysis

**Figure 15:**
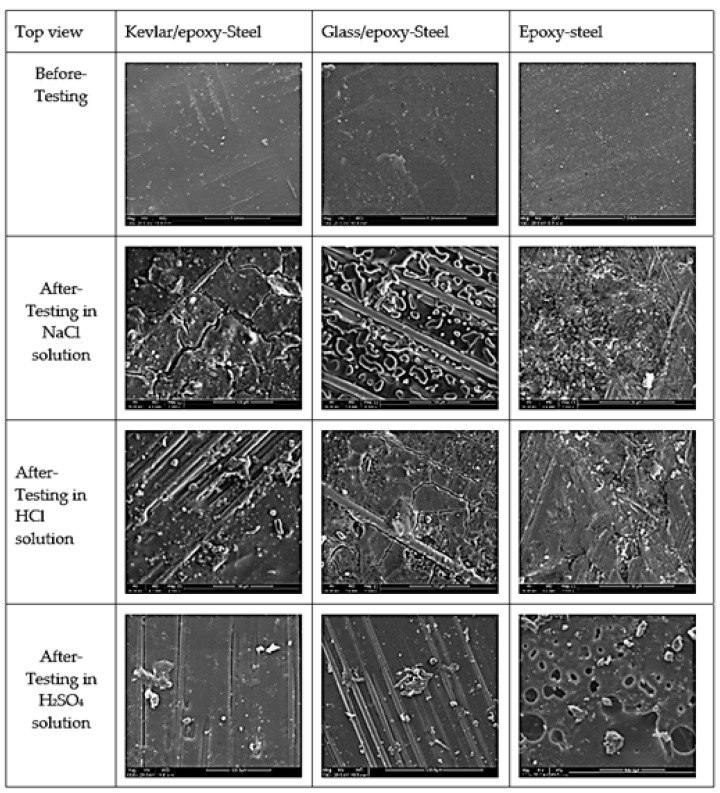
SEM images of the tested samples before and after testing.

The SEM images presented in Figure 15 reveal the nature of the samples before and after testing from a top view. They reveal cracks and the exposure of the embedded fibers from under the epoxy subjected to deterioration from acid attack, sustained/assisted by the diffusion of corrosion species. Similar findings have been reported by many researchers [27,50,51,52], who have stated that glass and Kevlar fiber-reinforced polymer composites degrade when subjected to environmental aging. However, the attack was more noticeable in the case of KFRP, since the greater degree of degradation in the fiber and fiber/matrix interface was recognized because of the greater moisture uptake of the Kevlar material. In contrast, glass fibers have a lower susceptibility to moisture [53].

## 4. Conclusions

The results of this comprehensive study indicate the high reliability of the coating systems involving glass, Kevlar, and epoxy against the corrosion of the carbon steel substrate in the various corrosive solutions studied: 0.5 M NaCl, 0.5 M HCl, and 0.5 M H_2_SO_4_. Generally, the GFRP coating system offered the best protection, with a high stability against deterioration, promoted by the glass fiber and epoxy compared with the epoxy and KFRP coating systems. The following conclusions can be drawn:

- As demonstrated by the open circuit potential results, the Kevlar/epoxy system interestingly showed deterioration in the H_2_SO_4_ solution comparable to an exposed substrate after nearly 50 ks.
- The corrosion rates for the coated substrates were 1 million to 10 million times less than that of the exposed substrates, as reported by the PDP tests.
- The equivalent circuit of the configuration, {R(Q(R(QR)))}, was correctly fitted to the experimental data across the entire frequency range.
- The peaks of the glass/epoxy and epoxy coating systems in the Bode diagrams indicated reliability against activity at both the double and coating layers, appearing at frequencies two orders of magnitude higher than those of the Kevlar/epoxy system.
- The resistance of the glass/epoxy system in the HCl solution was the highest, although it showed a steady decrease with time. The Kevlar/epoxy system, on the contrary, had less resistance, but it noticeably increased with time. This result underlines the significance of Kevlar in promoting the formation of passive films that, with time, decelerate dissolution and confirm the significance of glass in stabilizing the epoxy system that initially protects the system.
- The SEM images revealed cracks and the exposure of the embedded fibers from under the epoxy subjected to deterioration from the acid attack, sustained/assisted by the diffusion of the corrosion species.

## Figures and Tables

**Table 1:** Comparison study on the electrochemical behavior of different coating systems for CS.

Refs.	Year	Coating System	Immersion Period	Soln.	Test	Behavior
[37]	2009	Undoped polyaniline epoxy blend coating	300 days	NaCl	OCP and EIS	Coating resistance decreased at day 14 and then stabilized during the remaining immersion time.
[38]	2020	CFRP	42 days	Seawater	Anodic polarization and EIS	Carbon fiber degraded under the action of corrosive medium and polarization due to the corrosion damage of resin matrix, carbon fiber, and interface.
[39]	2019	CFRP and GFRP	48 h	NaCl	Electrochemical method (climate accelerated process)	The corrosion rates of carbon-type and glass-type were less than 1/10 and 1/100, respectively, than that of an ordinary steel bar.
[40]	2020	Epoxy primer and Polyurethane acrylic paint	98 days	Seawater	OCP, linear polarization, and EIS	A loss of thickness over time was recorded.
[41]	2021	sulfur epoxy resin SER/HMDA and SER/MDA composite polymeric coatings	24 h	NaCl	PDP and EIS	Composite polymers could retard and chloride ions attack.
[42]	2020	CFRP	8 h	NaCl+ CaCl_2_+ NaHCO_3_	OCP, PDP, and EIS	CFRP can cause galvanic corrosion to engineering metal in an aggressive environment.
Current Study	--	GFRP, KFRP, and pure epoxy	25 h	NaCl, HCl, and H_2_SO_4_	OCP, PDP, and EIS	Results and conclusions are discussed in the following sections.

**Table 2:** Chemical composition of the EN10130 carbon steel sheet.

Fe	C	Mn	P	S	Si
99.09	0.12	0.6	0.045	0.045	0.1

## Data Availability

The data presented in this study are available on request from the corresponding author.
